# Microbial Hydrolysates as Amino Acid Source in Cell Culture Media for Cellular Agriculture

**DOI:** 10.1002/bit.70290

**Published:** 2026-07-03

**Authors:** Jelle C. Hoek, Jean‐Marc Daran, Marieke E. Klijn, Marcel A. Vieira‐Lara

**Affiliations:** ^1^ Department of Biotechnology Delft University of Technology Delft the Netherlands

**Keywords:** amino acids, cost reduction strategies, Cultivated meat, cultivation media, medium optimization, microbial hydrolysates

## Abstract

The high cost of cultivation media remains one of the barriers to the commercialization of cultivated meat. Current serum‐free media reduce reliance on fetal bovine serum but only achieve modest cost savings. Especially at larger scales (> 20 m^3^), pharmaceutical‐grade amino acids and recombinant proteins remain major cost drivers. Techno‐economic analyses suggest that replacing purified amino acids is essential to achieve price parity with conventional meat. Microbial hydrolysates, also referred to as extracts, offer a promising alternative, as these can supply complex mixtures of amino acids and peptides at lower cost and with greater scalability than chemically defined formulations. In particular, yeast and bacterial extracts combine high protein (and therefore high amino acid) content, rapid growth, and established industrial‐scale production. Comparative analyses indicate that microbial amino acid profiles broadly overlap with those of animal cells and traditional meat, but this similarity does not translate into meeting cellular amino acid demand. Consumption data indicate that key amino acids, such as glutamine, cysteine, serine and arginine, would be supplied insufficiently by microbial extracts. This mismatch highlights the need for engineering of microbial biomass and optimization of extraction methods. Extraction methods such as autolysis, enzymatic lysis, or physical disruption strongly influence nutrient release and composition, underscoring the need for standardizing microbial extract preparation. In addition, challenges remain in ensuring consistency, safety, and bioavailability of nutrients, as microbial extracts also contain nucleic acids and potential toxins. This review summarizes current knowledge on microbial hydrolysates for cultivated meat media, including biomass composition, extraction methods, and analytical tools to assess quality and performance. We identify key knowledge gaps, particularly in quantitative amino acid consumption data for relevant cell lines and performance testing in scalable cultures. Addressing these gaps could enable cost‐effective media development and accelerate research toward sustainable, commercially viable cultivated meat.

## 1 Introduction

The world population is expected to grow to nearly 10 billion people by 2050, leading to an enormous demand for food products from livestock food systems (World Resources Institute 2019). Since food systems account for approximately one‐third of global greenhouse gas (GHG) emissions, low‐emission alternatives are urgently needed (Crippa et al. 2021). Cellular agriculture offers a promising solution by producing animal products through tissue and bioprocess engineering in controlled environments. Cultivated meat addresses many of the ethical and environmental issues associated with conventional livestock production, as it avoids animal slaughter and significantly reduces land use, GHG emissions, and water consumption (Tuomisto et al. 2024). While it has potential as a sustainable alternative to livestock products, the high production cost of cultivated meat remains a major challenge (Tuomisto et al. 2024). One of the most significant cost drivers is the growth medium required for cell proliferation (Hubalek et al. 2022).

Industrial‐scale animal cell cultivation is performed in the biopharmaceutical sector, primarily using mammalian cell lines such as Chinese Hamster Ovary (CHO) and Human Embryonic Kidney lines (Dumont et al. 2015). For biopharmaceutical applications, DMEM/F‐12 is one of the most widely used basal growth media, typically containing glucose, amino acids, vitamins, inorganic salts, and other components, and is often supplemented with fetal bovine serum (FBS). However, FBS is unsuitable for large‐scale production of cultivated meat, as it is not animal‐slaughter free, shows batch‐to‐batch variability, and is prohibitively expensive, hindering upscaling (Post et al. 2020). To overcome these challenges, several chemically defined FBS‐free media were developed, including Essential 8™, B8 (Kuo et al. 2020), Beefy‐9 (i.e., Beefy‐8 with recombinant albumin) (Stout et al. 2022). While these formulations eliminate FBS, they still make use of expensive recombinant proteins, such as fibroblast growth factor 2, albumin, insulin or insulin‐like‐growth factor 1, transferrin, transforming growth factor β, or activin‐A. Although these proteins are required only in low concentrations, they contribute disproportionally to medium cost due to their pharmaceutical‐grade purity and limited production scale. For example, nearly 98% of costs in the chemically defined B8 medium are derived from growth factors FGF‐2 and TGFβ (Quek et al. 2024).

At bench‐scale, FBS‐supplemented medium costs around $290/L (US dollars per liter), while a chemically defined alternative, such as Beefy‐9, costs around $217/L (Stout et al. 2022). Consequently, research has continued to focus on reducing the cost contribution of recombinant proteins. For example, Beefy‐R medium builds on FBS‐free Beefy‐9 medium by replacing albumin with rapeseed protein isolate (RPI). Since the processing costs of RPI are not known, the raw material cost contributions are used in this comparison. Recombinant albumin contributes $24.56 per liter medium while RPI only contributes $0.002 per liter (Stout et al. 2023). This reflects a potential component‐level reduction of several orders of magnitude.

However, techno‐economic analyses (TEAs) demonstrated that replacing FBS by growth factors and other recombinant proteins alone is not sufficient to reduce costs to a level that would enable market entry of cultivated meat (Goodwin et al. 2024). A cost breakdown of an augmented Beefy‐R medium for large‐scale production (42 to 262 m^3^, 100 g cells/L) reveals that growth factors would account for 24% of the total costs, while amino acids would account for 76% (Negulescu et al. 2023, Table S1). Similar to recombinant proteins, the high cost for amino acids stems from their current low production scale and the requirement for high‐purity pharmaceutical‐grade products (Humbird 2021). Moreover, the energy demand for pharmaceutical‐grade products is estimated to be 20 times higher and the global warming potential 25 times greater than bulk chemical production (Risner et al. 2025). It is expected that the amino acid market will likely adapt to meet the demands of the cultivated meat industry, with prices converging toward those of amino acids already produced for animal feed (Humbird 2021). For example, lysine, methionine, and threonine are currently produced in large volumes (> 10 kiloton per year), due to their role as limiting amino acids in animal feed formulations. In contrast, amino acids such as histidine, serine, and tyrosine (< 1 kiloton per year) are primarily used in pharmaceutical applications and thus remain more costly.

An emerging strategy to reach cost‐effective amino acid supply is the use of complex hydrolysates as alternative amino acid sources (Flaibam et al. 2024). Examples include plant hydrolysates, such as soy and rapeseed, and microorganism hydrolysates, such as algal, bacterial, and fungal hydrolysates. While plant hydrolysates are inexpensive and broadly available, they present challenges such as batch‐to‐batch variability, allergenicity risks (e.g., soy, pea, and wheat), and dependence on seasonal agricultural supply (Mi et al. 2025). Microbial hydrolysates are inexpensive to produce, offer prospects for sustainable and scalable production, and generally have a higher protein content (~50%‐80% dry weight depending on species) compared to plant protein sources such as soy (~30%‐40%) (Muazzam et al. 2025; Onyeaka et al. 2022). Medium supplementation with microbial hydrolysates has been shown to support the proliferation of different mammalian cell lines (Hu et al. 2018; Spearman et al. 2016). Additionally, microbial strains could be engineered to produce customizable nutrient profiles (Tao et al. 2023). While plants also allow genetic engineering, microbial systems enable faster design‐build‐test cycles due to their shorter generation times and simpler genetic manipulation.

To date, studies using microbial hydrolysates primarily focused on FBS replacement in small‐scale 2D (adherent) culturing systems (Table 1), most commonly using extracts derived from *Saccharomyces cerevisiae* (*S. cerevisiae*, also known as baker's yeast). While replacing FBS in the medium is crucial, replacing amino acids in growth medium is still largely unexplored despite the potential economic gain (Goodwin et al. 2024). Using ≥ 2 m^3^ bioreactors is often recommended for an economically viable cultivated meat product, and bioreactors at this scale only use suspension‐adapted cell lines (Goodwin et al. 2024; O'Flaherty et al. 2020). An optimized medium composition should, therefore, also be tested in suspension culturing systems, to determine industrial‐scale applicability. Moreover, microbial extracts intended for cultivated meat production should be able to replace amino acids without inhibiting cell growth, must be food‐grade or at least nontoxic for human ingestion, and should avoid contaminants such as microbial toxins that are difficult to remove during downstream processing (Lobo‐Alfonso et al. 2008).

**Table 1:** Overview of microorganism hydrolysates used in the literature to promote animal cell growth.

Animal cell line	Microorganism used for extract	Extraction method	Medium	Supplementation	Goal	Main conclusions	Source
3D CHO	*Saccharomyces cerevisiae* (*S. cerevisiae*)	Commercial: HyPep™ YE (Kerry Group, Almere, NL)	Protein free and chemically defined medium	5% (w/v)	Promote growth & mAb production	Extract promotes cell growth, increases cell size, and increases recombinant protein productivity compared to control.	Hu et al. (2018)
2D primary skeletal muscle cells	*S. cerevisiae*	Commercial: Yeast Extract (Duchefa Biochemie, Haarlem, NL, cat: Y1333)	DMEM with and without 2% FBS	0.00001%–1% (w/v)	FBS replacement or cost reduction	Extract is added in serum‐free media and recovers growth rate to serum‐containing control.	Andreassen et al. (2020)
2D C2C12 mouse myoblasts	*Chlorococcum littorale, Stichococcus sp., Chlorella vulgaris, Euglena gracilis, Spirulina subsalsa, Arthrospira platensis*	Acid/alkali hydrolysis	DMEM	5%–20% (v/v)	Glucose and nutrient replacement in media	Improved single passage proliferation compared to DMEM without FBS	Okamoto et al. (2020)
2D primary bovine myoblasts	*Chlorella vulgaris* (algae)	Acid/alkali hydrolysis	Inorganic salt solution + 10% FBS	5% (v/v)	Glucose and nutrient replacement in media	Similar proliferation with algal supplementation as DMEM, both + FBS	Okamoto et al. (2022)
2D and 3D CHO and mesenchymal stem cells	*C. vulgaris*	Powder grinding and boiling	DMEM + 10% FBS	0.0001% (w/v)	FBS/growth factor replacement	Improved single passage proliferation in 0, 5, and 10% FBS conditions with extract	Ng et al. (2020)
2D human lung carcinoma cell line H460	*Spirulina maxima* (cyanobacterium)	Sonication and high temperature	RPMI 1640 + 0–10% FBS	0%–3% (v/v)	FBS replacement	No toxicity with ≤ 2% addition, cell growth similar to control at 50–70% FBS replacement	Jeong et al. (2021)
2D CHO	*S. cerevisiae*	French press + autolysis	Serum‐free medium	0–7.5% (v/v)	Promote growth & mAb production	Analysis of extract helped formulate chemically defined medium to support similar proliferation as extract	Spearman et al. (2016)
2D porcine muscle satellite cells	*S. cerevisiae*	Bead beating	DMEM + 5% FBS	0.1%–0.5% (w/v)	FBS replacement	Extract‐supplemented medium with recombinantly expressed cytokines replaces commercial cytokines in 5% FBS	Lei et al. (2023)
2D immortalized bovine satellite cells	*Vibrio natriegens, Lactiplantibacillus plantarum, Bacillus subtilis, Lactococcus lactis, S. cerevisiae*	Sonication	Serum free medium (B8)	0.0001%–0.004% (w/v)	Recombinant albumin replacement	Extract replaces recombinant albumin with similar growth to 20% FBS after adaptation of the cell line to the new medium	Dolgin et al. (2025)
2D and 3D (microcarrier) immortalized goldfish muscle cells	*Auxenochlorella pyrenoidosa*	Heat lysis (121°C)	Basal medium + 5% FBS	0.01%–0.1% (w/v)	FBS replacement	Extracts decrease FBS supplementation in medium from 10% to 5%, and promote adherence to microcarriers in 2% FBS	Dong et al. (2023)

*Note:* 2D and 3D Indicate That Adherent or Suspension Cell Lines Were Used, Respectively.

*Source:* Table Adapted and Expanded From (Fan et al. 2024), (Du et al. 2022), and (Y. Y. Ho et al. 2021).

Abbreviations: B8: Chemically Defined Medium Optimized for Human Induced Pluripotent Cells; CHO: Chinese Hamster Ovary cells; DMEM: Dulbecco's Modified Eagle's Medium; FBS: Fetal Bovine Serum; mAb: Monoclonal Antibody; RPMI 1640: Roswell Park Memorial Institute 1640 Medium; v/v: Volume Per Volume; w/v: Weight Per Volume; YE: Yeast Extract.

A microbial extract capable of replacing pharmaceutical‐grade amino acids in cultivated meat media could dramatically reduce overall production costs and accelerate commercialization. This review outlines the requirements for microbial hydrolysates as amino acid replacement in cultivated meat media, with a focus on animal cell physiology and bioprocess considerations. Techno‐economic and regulatory considerations, while crucial for the translation of this technology, are beyond the scope of this review and have been addressed elsewhere (Goodwin et al. 2024; Pasitka et al. 2024; Risner et al. 2025). We summarize the current knowledge on microbial extracts used in animal cell culture and identify current knowledge gaps. Specifically, we compare animal cell amino acid requirements with microbial biomass composition and discuss species selection and extraction methods. Finally, we highlight approaches to test microbial extracts as amino acid replacement in relevant culture systems.

## 2 Amino Acids

### 2.1 Amino Acid Requirements for Animal Cells

A commonly used basal medium, DMEM/F‐12, can support the growth of a wide range of animal cell lines. It is a 1:1 mixture of DMEM and Ham's F‐12 medium, where it combines the high glucose and amino acid concentrations of DMEM with the wide variety of components from Ham's F‐12 medium. The high glucose concentration exacerbates the Warburg effect, leading to accumulation of lactate, which begins to inhibit growth at 10–20 mM (Hosios et al. 2016). Amino acids are the major contributors to cell biomass, and the most abundant amino acid in DMEM/F‐12, glutamine (2.5 mM), is used as an energy, carbon, and nitrogen source (Hosios et al. 2016). Glutamine is incorporated into biomass by replenishing tricarboxylic acid cycle intermediates after being deaminated, making it an important anaplerotic substrate for the cycle (Deberardinis and Cheng 2010). However, high metabolization of glutamine leads to production of ammonium, which starts inhibiting growth at 1–4 mM (Cruz et al. 2000). Next to the high concentrations of glucose and glutamine, there are 49 additional components in the DMEM/F‐12 basal medium. This results in a nutrient‐dense and expensive medium, not optimized for specific cell lines. Optimization of the DMEM/F‐12 medium for human induced pluripotent stem cells (hiPSCs) using a titration assay led to a reduction in medium complexity, down to just 39 components (Lyra‐Leite et al. 2023). Nevertheless, even in this optimized medium, 46% of the costs of this basal medium still arise from the amino acids (for calculation see Supporting Information S1: Section 10.1).

Amino acids are traditionally classified as essential (His, Ile, Leu, Lys, Met, Phe, Thr, Trp, and Val) or non‐essential (Ala, Arg, Asn, Asp, Cys, Glu, Gln, Gly, Pro, Ser, and Tyr), depending on whether animal cells can synthesize the amino acid de novo. However, this distinction is not absolute. Mammals such as humans, pigs, cattle, and sheep can synthesize arginine from glutamic acid, glutamine and proline, but still show physiological symptoms under arginine deficiency (Hou and Wu 2018). Additionally, arginine is often required during fast growth or stress and is therefore regularly categorized as semi‐essential (Tong and Barbul 2004). In vitro, this conventional classification can be misleading. For example, arginine, cysteine, glutamine, and tyrosine are essential for hiPSCs, while the traditional classification is non‐essential (Lyra‐Leite et al. 2023). Nutrient utilization also varies by cell type and stage; rapid depletion of glutamine and serine was reported in C2C12 myoblasts and fibroblasts, while leucine and valine remained stable, and alanine and proline accumulated (O'Neill et al. 2022). Moreover, amino acid metabolism and consumption behavior change during cell differentiation. For example, alanine metabolism shifts from secretion during proliferation to consumption during differentiation in C2C12 myoblast cells (O'Neill et al. 2021).

Calculations of anabolic amino acid requirements of cells could be in principle derived from whole‐cell amino acid composition from total protein content. However, these whole‐cell‐derived anabolic requirements do not align with the actual amino acid consumption profile of cells (Széliová et al. 2020). In this study using CHO cells as model, glutamine consumption was circa 10 times higher than strictly required for protein biosynthesis, depending on the evaluated condition, which is consistent with its anaplerotic role. The consumption rates of other amino acids, such as asparagine and serine, were higher than would be predicted based solely on biomass composition. Glutamate and aspartate were secreted by cells because of glutamine and asparagine deamination, respectively. In addition, alanine and glycine were reported to be produced by cells in all experimental setups, aligning with previous studies in other animal cell lines (Hosios et al. 2016; Vriezen 1998). Overall, the difference between predicted and observed amino acid consumption stems from amino acid interconversion and further non‐proteinogenic metabolic roles. For example, the major source of glutamate in cell culture is glutamine due to deamination, and not free glutamate in the cell medium. Glutamine is also used as a major nitrogen source, meaning that it can donate its nitrogen for the synthesis of other compounds, such as nucleotides (Deberardinis and Cheng 2010), and serine can be a precursor for intracellular glycine. Amino acids are precursors for several metabolites in the cell, such as branched‐chain amino acids that can feed the TCA cycle (Neinast et al. 2019), and serine that is used for the biosynthesis of lipid headgroups (e.g., phosphatidylserine) (Handzlik and Metallo 2023).

As amino acid requirements are cell line and stage‐dependent, it is important to have data available for cellular agriculture‐relevant cell types. To date, amino acid depletion time courses have been reported for hiPSCs spent media (Lyra‐Leite et al. 2023), as well as for chicken muscle progenitors, chicken muscle fibroblasts, and C2C12 murine muscle cells (O'Neill et al. 2022). These datasets supported the development of a simplified basal medium for iPSCs and showed that several amino acids remain stable over time. However, others appeared to be partially depleted, namely the essential amino acids arginine, isoleucine, leucine and methioinine, as well as the non‐essential amino acids glutamine and serine. In addition, it was reported that a total of 252.3 g of amino acids is required to produce 1 kg wet weight C2C12 murine muscle cells (Negulescu et al. 2023). This calculation is based on a cell weight of 3 ng/cell for C2C12 murine muscle cells and is thus highly dependent on accurate determination of the cell weight. A generally accepted animal cell wet weight ranges from 3 to 4 ng/cell, but it may vary from 1 to 5 ng/cell (Humbird 2021). Different growth conditions and growth phase (exponential or stationary) may impact this estimate. For example, CHO cells can have a threefold increase in average volume and dry weight per cell over the course of a bioprocess (Pan et al. 2017). Therefore, from a bioprocess perspective, it is preferred to report amino acid requirements per dry weight. This is a necessary unit to estimate medium requirements in industrial‐scale suspension cultures (Pan et al. 2017; Széliová et al. 2020; Vriezen 1998) as this unit controls for cell size, which poorly correlates with dry weight and water content (Széliová et al. 2020). Tailored medium formulations based on actual cellular requirements normalized by dry weight will be essential to enable comparisons across cell lines and process conditions, focusing on decreasing costs and improving industrial feasibility.

### 2.2 Amino Acid Composition of Animal Cells

**Figure 1:**
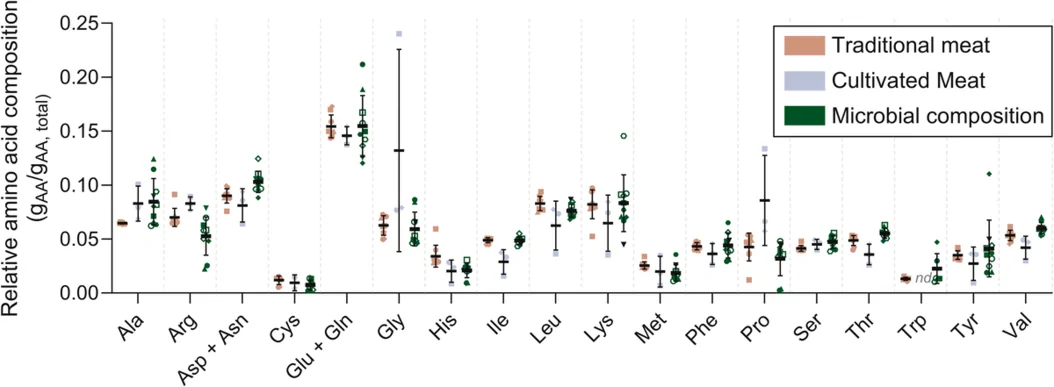
Ranges of amino acid ratios of traditional meat (red floating bars), cultivated meat (blue floating bars) and microbes (green scattered symbols). Traditional meat amino acid data are from beef (●), pork (■), lamb (▲), and chicken (◆) (*n* = 9), and cultivated meat data are from pork, chicken, and beef primary muscle skeletal cells (*n* = 3, same symbols as traditional meat) (Anaeto et al. 2010; Dida 2022; Joo et al. 2022; Kim et al. 2025). Floating bars show the minimum to the maximum data, with a line at the mean. Microbial amino acid fraction shows individual points (*n* = 10), with a line representing the mean, and the error bar the standard deviation of the mean. “nd” denotes cases where data were not available. The datapoints represent the following organisms: *Saccharomyces cerevisiae* 1 (●) and 2 (■) (Anderson et al. 2024; Demirgül et al. 2022), *Kluyveromyces marxianus* (▲) (Demirgül et al. 2022), *Yarrowia lipolytica* (◆) (Jach et al. 2017), *Cyberlindnera jadinii* 1 (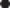) and 2 (○) (Anderson et al. 2024; Lapeña et al. 2020), *Candida tropicalis* (□) (Pessoa 1996), *Bacillus subtilis* (▼) (Kurbanoglu and Algur 2002), *Pichia kudriavzevii* (◇) (Ohlsson et al. 2019), *Corynebacterium glutamicum* (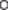) (Zhang et al. 2013). Asp + Asn and Glu + Gln are grouped because many cell lysis methods convert Asn and Gln into Asp and Glu, respectively. Data of all individual data points (traditional meat, cultivated meat, and microorganisms) are available in tab 2 of Supporting Information S2. Cultivated meat amino acid values were compared with traditional meat and microbial composition.

While understanding the amino acid requirements of animal cells is essential for medium optimization, the amino acid composition of animal cell biomass can provide insights into the nutritional profile of the envisioned product. As cultivated meat aims to replicate conventional meat, compositional profiles are typically compared to the tissue composition of traditional meat, and reported ranges largely overlap (Figure 1). Variability across studies reflects differences in medium composition, differentiation stage, and product formulation (Joo et al. 2022; Kim et al. 2025). For example, Joo et al. reported differentiated satellite cells from cow and chicken muscle tissue, adherently grown in a 2D layer (Joo et al. 2022), while Kim et al. studied differentiated pig muscle stem cells, grown in a 3D scaffold (Kim et al. 2025). In both examples, a basal medium with an addition of 10% to 30% FBS was used (Joo et al. 2022; Kim et al. 2025). Notably, cultivated meat shows 69% lower levels of isoleucine (*p* < 0.001) when compared to traditional meat.

### 2.3 Amino Acid Composition of Microorganisms

**Figure 2:**
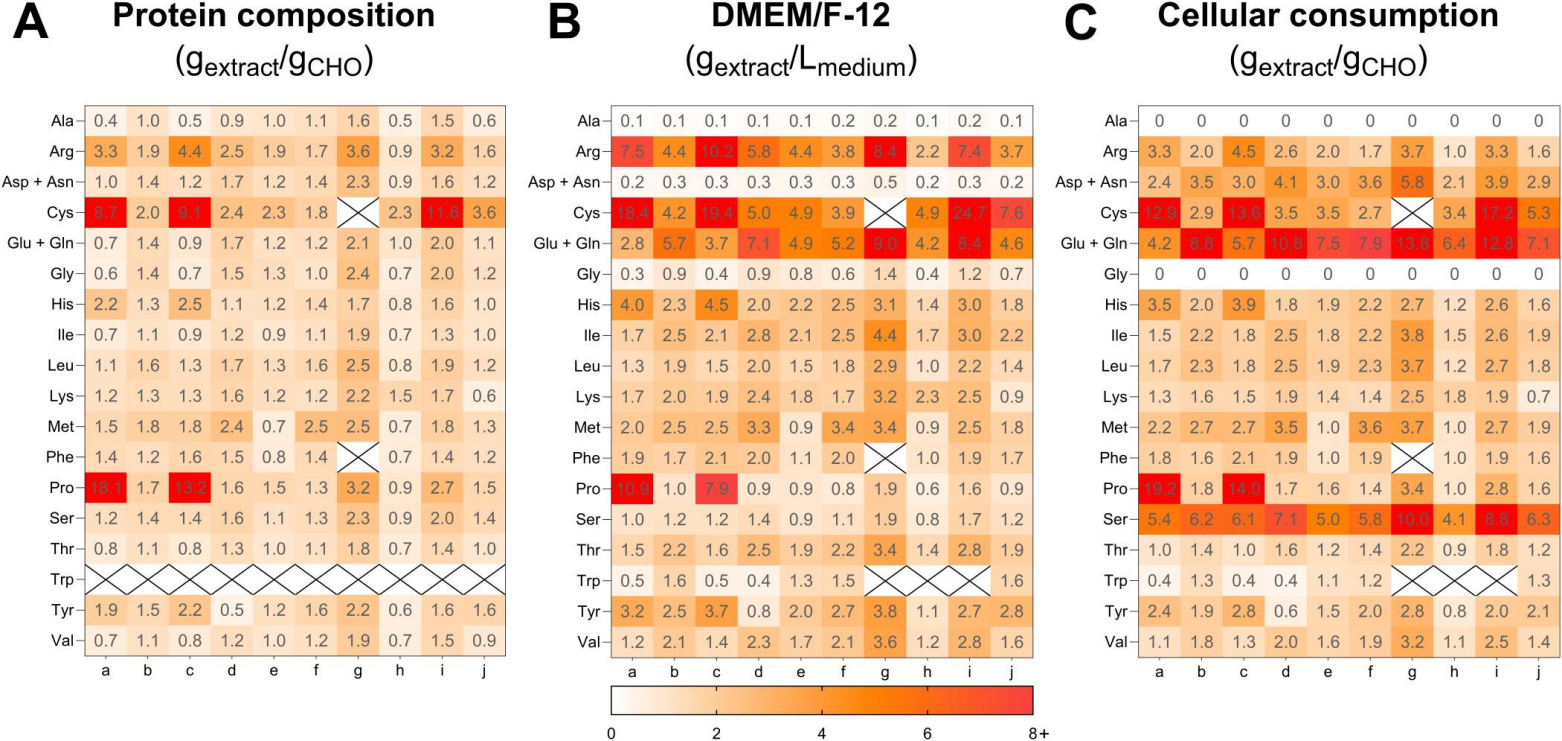
Amount of microbial extracts needed to supplement amino acids based on CHO cell amino acid composition (A), DMEM/F‐12 composition (B), and consumption rates of CHO cells (C). For each comparison, the values shown represent grams of extract (g_extract_) required to supplement the amino acid need for either a gram of CHO cells in dry weight (g_CHO_) or a liter of medium (L_medium_). Comparisons are based on an average protein composition of 0.5 g_prot_/g_CHO_ for CHO cells and 0.5 g_prot_/g_extract_ for microbial hydrolysates. For (A), the calculation was based on (g_AA_/g_CHO_)/(g_AA_/g_extract_), for (B) on (g_AA_/L_medium_)/(g_AA_/g_extract_) and for (C) on on (q_AA_/µ)/(g_AA_/g_CHO_), with q_AA_ (biomass‐specific amino acid uptake rate) in g_AA_/(g_CHO_ ⋅ h) and µ (growth rate) in 1/h. Columns show data per organism: a: *Saccharomyces cerevisiae* (Demirgül et al. 2022), b: *Saccharomyces cerevisiae* (Anderson et al. 2024), c: *Kluyveromyces marxianus* (Demirgül et al. 2022), d: *Yarrowia lipolytica* (Jach et al. 2017), e: *Cyberlindnera jadinii* (Anderson et al. 2024), f: *Cyberlindnera jadinii* (Lapeña et al. 2020), g: *Candida tropicalis* (Pessoa 1996), h: *Bacillus subtilis* (Kurbanoglu and Algur 2002), i: *Pichia kudriavzevii* (Ohlsson et al. 2019), j: *Corynebacterium glutamicum* (Zhang et al. 2013). Data that were not available are marked with an X. A zero amino acid requirement means it is produced by the cells. Asp + Asn and Glu + Gln are grouped since many cell lysis methods convert Asn and Gln into Asp and Glu, respectively. Full calculation of the data is available in tab 4 of Supporting Information S2.

Comparing the amino acid composition of several microbial extracts with traditional and cultivated meat shows that amino acid levels are mostly within the same range, with highly similar profiles across different microorganisms and only minor quantitative differences (Figure 1, Supporting Information S1 : Section 10.3). Interestingly, the average amino acid distribution of microbial extracts more closely resembles traditional meat than cultivated meat (Figure 1). Statistical comparison revealed that, among all amino acids, only isoleucine, threonine, and valine differed significantly between cultivated meat and microbial extracts (*p* < 0.01). Humbird conducted a similar analysis, with CHO cell composition as a proxy for cultivated meat and soybean hydrolysate instead of microbial extract. 1.36 moles of soybean hydrolysate were needed to meet composition‐based amino acid requirements of 1 mole of CHO cells, except for glutamine, tyrosine, alanine and asparagine (Humbird 2021, Figure 2). To cover all amino acid requirements, this estimate increases to 5.57 moles of soybean hydrolysate per 1 mole of CHO cells. Comparable calculations for hydrolysates from *S. cerevisiae* (Anderson et al. 2024), *Cyberlindnera jadinii* (*C. jadinii*, formerly *Candida utilis*) (Lapeña et al. 2020), and *Bacillus subtilis* (*B. subtilis*) (Kurbanoglu and Algur 2002) show that 1.49, 1.60, and 2.91 moles are required, respectively, to fulfill essential and non‐essential amino acid needs of CHO cells (see Supporting Information S1: Section 10.2). The soybean hydrolysate was matched based on threonine (limiting amino acid), and all microorganism extracts were matched based on cysteine, which often has the lowest concentration in microbial extracts. We adapted and expanded this analysis to calculate the required amount of hydrolysate needed for animal cells based on either (A) animal cell biomass composition, (B) standard cell culture medium composition, or (C) experimental amino acid consumption data (Figure 2). In this analysis, we use CHO cell data as a proxy for cultivated meat cells, given the availability of quantitative amino acid consumption data in suspension CHO cultures without FBS, and the lack of such information for cultured cells relevant for cellular agriculture.

As expected, none of ten microorganisms (8 yeasts and 2 bacteria) perfectly match the amino acid composition of CHO cells, their consumption profile, or the formulation of DMEM/F‐12. Tailoring extract supplementation based on a single amino acid produces a surplus of others, potentially inhibiting growth at high concentrations. For example, medium concentrations below 0.5–1 mM are recommended for glycine, leucine, methionine, and phenylalanine in CHO cell medium (Pereira et al. 2018). Additionally, maintaining amino acids outside the low range (0.5–1 mM) increases the accumulation of metabolic byproducts that inhibit growth in human embryonic kidney cells (Harrington and Mulukutla 2025). Moreover, variability exists between extracts from the same species (e.g., *S. cerevisiae*; Supporting Information S1: Section 10.4), underscoring the need for standardized production protocols. Hydrolysates from protein‐rich organisms are needed in lower amounts than hydrolysates from protein‐poor organisms given their higher amino acid content. For example, less extract is required from a *Bacilus subtilis* extract (organism h) than from *Pichia kudriavzevii* (organism i) (Figure 2). This highlights protein content of the microorganism as an important selection criterion.

The comparison of microbial biomass composition and the biomass composition of CHO cells shows that a high amount of microbial extracts would be needed to supply enough arginine, cysteine, and proline (Figure 2A). The high requirements for proline might originate from the high variation in proline content in the microbial composition dataset. Taking the diversity of microbial extracts into account, a median value of 3.0 g_extract_ (dry weight) is needed to fulfill all essential and non‐essential amino acid needs to produce 1 g_CHO_ (dry weight). However, as previously addressed in Section 2.1, the amino acid composition of animal cells differs from the actual amino acid demand and should not be used to guide medium development. A commonly used intermediate approximation is the amino acid composition of growth media such as DMEM/F‐12, which still reflects historical formulation choices rather than quantified cellular demand. When compared to DMEM/F‐12, high amounts of microbial extracts would be needed to supply arginine, cysteine, and glutamate/glutamine (Figure 2B), indicating these are the most limiting amino acids in microbial biomass. When compared to CHO cell consumption rates, glutamine/glutamate and serine emerge as main limiting amino acids in microbial hydrolysates (Figure 2C). This yields a median value of 10.0 g_extract_/g_CHO_ to fulfill all amino acid needs based on consumption data, and 5.0 g_extract_/g_CHO_ if glutamine and serine are excluded, indicating that these two amino acids might have to be supplemented in the final growth medium. It should be noted that these values depend on culture conditions, given that serine, like glycine and glutamine, can be omitted from the growth medium without completely halting proliferation, highlighting the adaptability of cultured animal cells (Hosios et al. 2016; Széliová et al. 2020). This analysis shows how the choice of reference framework (amino acid composition, DMEM formulation, or amino acid uptake rates) profoundly affects the estimates of microbial extract requirements as amino acid source for animal cells. Amino acid consumption profiles, rather than amino acid composition of the biomass, should guide medium design. This analysis is based on CHO cell data given the absence, to our knowledge, of quantitative data of cultivated meat‐related cell lines. To guide medium development for cellular agriculture, flux data on amino acid demand for relevant cell lines is required (Gomez Romero et al. 2025). Furthermore, culturing animal cells under different regimes of single amino acid limitation will teach us about the physiological amino acid requirements for medium formulation.

## 3 Microorganisms

### 3.1 Protein Content and Microbial Toxins

Among microorganisms, bacteria typically have the highest protein content (> 60%–80% protein in dry weight), followed by yeast and fungi (40–60% dry weight) (Onyeaka et al. 2022). Much of this compositional data originates from single‐cell protein (SCP) research, which evaluates microbial biomass for its nutritional value in food and feed applications. Examples of bacterial hydrolysates include *Escherichia coli*, *B. subtilis*, and *Bacillus cereus*, with a protein content of 60%–70% (Kurbanoglu and Algur 2002). However, *B. cereus* is not suitable due to pathogenicity concerns and enterotoxins (Foodsafety.gov 2023; Jeßberger et al. 2014). Yeast extracts, particularly from *S. cerevisiae* and *Saccharomyces pastorianus* are the most widely used microbial extracts in both food and microbial media (e.g., Bacto Yeast Extract™, and spent brewers’ yeast, respectively). Fungi are also used for protein production, most notably mycoprotein (Quorn™, derived from *Fusarium venenatum*) (Wikandari et al. 2023).

Microbial hydrolysates are complex mixtures that consist of intracellular contents and cell wall material. Yeast cell wall consists mostly of fibrous β‑glucans (~60% by mass) and mannoprotein (~40%) with minor amounts of chitin (1%–3%) (Tao et al. 2023). These compounds, along with most insoluble membrane lipids, are often removed through centrifugation or other solid‐liquid separation when making hydrolysates. The rest of the cell contents consist of soluble amino acids, ribonucleotides, minerals, vitamins, and peptides (Tao et al. 2023). Bacterial cell walls differ: Gram‐positive species have a thick peptidoglycan (50% of cell wall mass) cell wall, whereas Gram‐negative bacteria have a much thinner wall (10%–20% of cell wall mass) (Allison and Lambert 2024). Next to that, Gram‐negative bacteria exhibit lipopolysaccharide on their outer membrane, an endotoxin that is difficult to remove from the final product (Lobo‐Alfonso et al. 2008).

**Figure 3:**
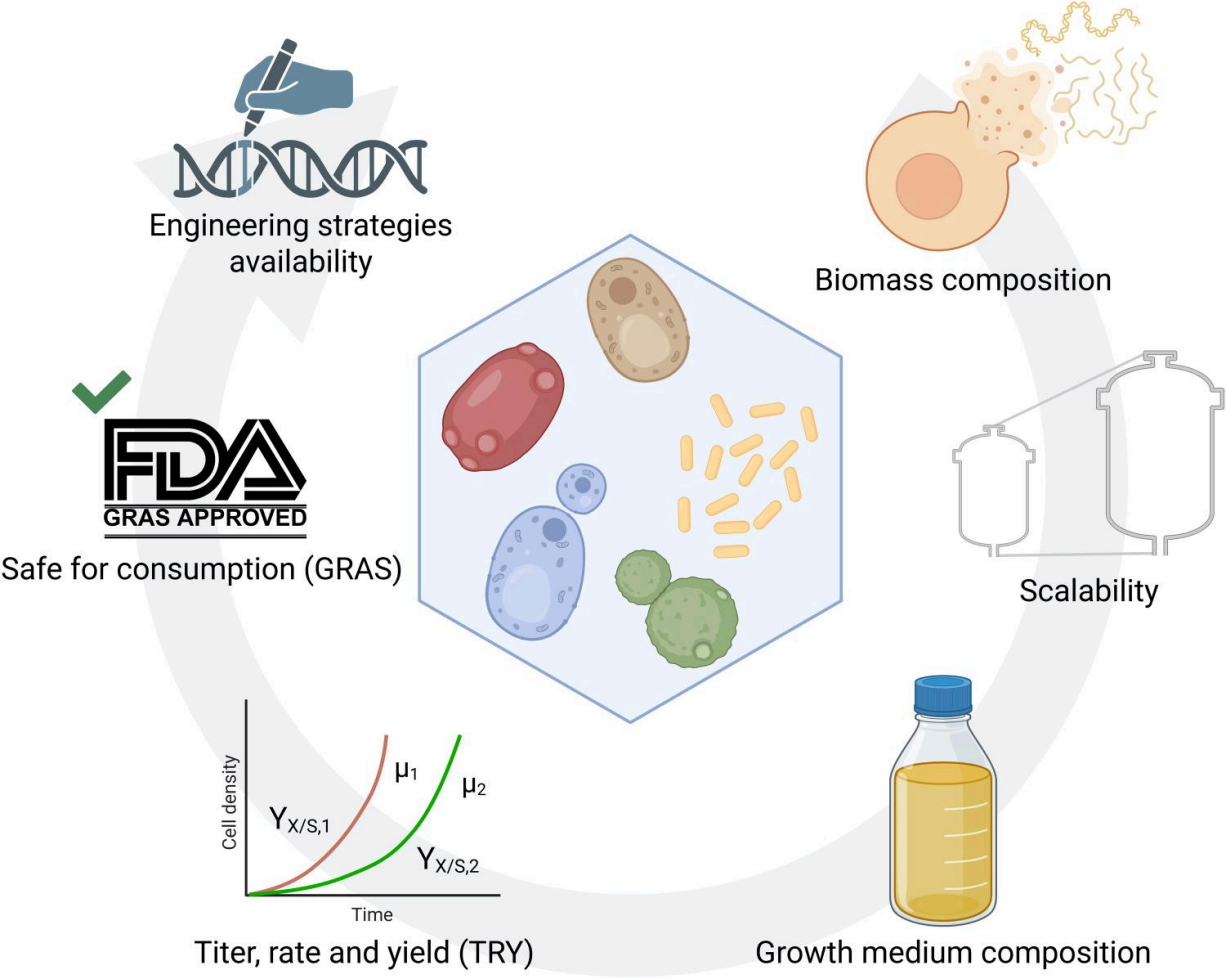
Microorganism selection criteria for production of an extract suitable for cultivated meat media. Created in Biorender.

Extracts also contain nucleic acids, up to 15%–16% in bacteria and 9% in fungi (Nangul and Bhatia 2013). Excess dietary nucleic acids can cause hyperuricemia and gout in humans (Podpora et al. 2016). Growth inhibition may occur if these concentrations exceed 0.35 mg/mL (1 mM) in CHO cells or 0.14 mg/mL in human cells (Carvalhal et al. 2003; Sawa et al. 2021). Minerals in the extract may increase the medium osmolality and thereby lead to osmotic shock in cultured cells. Medium optimization for hiPSCs showed values around 300 mOsm/L to be suitable for cell growth (Lyra‐Leite et al. 2023). When 5 g/L yeast extract was supplemented to CHO cell culture media, osmolality increased to 366 mOsm/kg, without affecting growth (S. Ho et al. 2016). While biomass composition is instrumental for assessing the suitability of microbial extracts, organism selection requires other factors, such as scalability, microbial growth medium composition, titer (cell density), rate and yield, safety for consumption or GRAS status (Generally Regarded As Safe), and engineering strategies availability (Figure 3).

### 3.2 Scalability

Global meat production in 2024 reached 365 million tonnes (or 3.65∙10^11^ kg) (OECD‐FAO Agricultural Outlook 2025–2034, 2025–2034 2025). Even a modest cultivated meat production target of 100 kilo tonne (1∙10^8^ kg, or 0.03% of traditional meat production) annually would require extract production at comparable scales (Humbird 2021, Section 3.1.1). Most used organisms in large‐scale industrial settings are bacteria, yeasts, fungi, algae, animal, and insect cell lines. For cost reduction of the final cultivated meat medium, raw material costs to produce the microbial extracts should be minimized. Animal and insect cells have slow doubling times (from 18 to more than 30 h (Abaandou et al. 2021; Saisud et al. 2023)) and require complex media with expensive, highly purified individual components and are therefore not desirable as raw materials for cultivated meat medium. Algae exhibit intermediate growth rates, with doubling times of 8 to 12 h (Tarin et al. 2021) and can convert light and CO_2_ into biomass. Although this could help in reducing net CO_2_ emissions in the cultivated meat industry, there are numerous challenges with scaling up algae growth, including contamination in outdoor environments and changing or insufficient light supply (Benner et al. 2022). The three organisms with the fastest doubling times are filamentous fungi (3–5 h), yeasts (1.5–2 h), and bacteria (20 min to several hours). Filamentous fungi, such as *Aspergillus* spp., present challenges due to variable macro morphologies such as pellets, clumps, or mycelia, hindering reproducibility (Meyer et al. 2021). Yeast production was estimated at 1540 kilo tonnes (1.54∙10^9^ kg) in 2017, indicating scalability with these organisms is feasible (Humbird 2021). Similarly, bacterial fermentation operates at multi‐kiloton scales in enzyme and amino acid production (Adrio and Demain 2014; Hirasawa and Shimizu 2016). Therefore, yeasts and bacteria stand out as the most suitable to enable production at the scales required for cultivated meat.

### 3.3 Microbial Growth Medium Composition

To ensure cost‐effectiveness, microbial biomass production should use simple and inexpensive media. Bacteria and yeasts can grow on synthetic, chemically defined media, consisting of a carbon source, inorganic salts, and vitamins if required by the organism (Demain 1958; Verduyn et al. 1992). Glucose is commonly used as a carbon source, and can be derived from corn, wheat, potato, or other food ingredients. Although it allows for more reproducible microbial biomass production when compared to more complex carbon sources (e.g., agricultural or food waste streams, which show batch‐to‐batch‐variability), it competes with food supply (Salim et al. 2019). Additionally, nitrogen fertilization, farm field operations, and processing also have a considerable environmental impact (Salim et al. 2019). Alternative substrates include food or agricultural waste streams (Mekureyaw et al. 2026), or C1, C2‐substrates (Almeida Benalcázar et al. 2025). Regarding nitrogen source, microbes can utilize simple molecules such as ammonia, which are cheaper to produce than glutamine (Demain 1958; Verduyn et al. 1992). Microbes consume these simple nitrogen molecules and convert them into biomass, including high‐value amino acids. Microorganisms capable of growing on simple, chemically defined media are therefore the most suitable candidates for the cost‐effective production of amino acids for cultivated meat medium.

### 3.4 Titer, Rate and Yield

The production cost of a bioprocess is largely determined by three key performance indicators: titer, rate, and yield (Konzock and Nielsen 2024). Batch mode (substrate excess, at maximum growth rate) can be run in bioreactors with controlled pH, but Crabtree‐positive yeasts such as *S. cerevisiae* usually show reduced biomass yield due to overflow metabolism (Anderson et al. 2024). In addition, biomass composition might differ over the course of a batch process, given its dynamic nature. A fed‐batch process is the preferred method for large‐scale production of biomass, given the controlled feed profile and ability to reach a high titer (Konzock and Nielsen 2024; Van Hoek et al. 2000; van Winden et al. 2022). For research, chemostats are preferred, given that cultivation is done at a defined and stable growth rate, producing biomass with reproducible characteristics (Anderson et al. 2024; Vieira‐Lara et al. 2024; Warmerdam et al. 2024). Reported biomass titers in fed‐batch cultures include up to 187 g/L cell dry weight for *S. cerevisiae* (Malairuang et al. 2020), 100 g/L for *C. jadinii* (Vieira‐Lara et al. 2024), 100 g/L for *Kluyveromyces marxianus* (*K. marxianus*), and 88 g/L for *B. subtilis* (Klausmann et al. 2021). These fermentation processes often start at a growth rate of 0.1–0.2 h^−1^, after which they switch from exponential to constant feeding to avoid oxygen limitation in the reactor. Biomass yields on glucose in fed‐batch cultures are reported as 0.50 g_x_g_s_^−1^ for *C. jadinii* (Anderson et al. 2024), 0.51 g_x_g_s_^−1^ for *K. marxianus* (Fonseca et al. 2007), and 0.25–0.30 g_x_g_s_^−1^ for *B. subtilis* (Klausmann et al. 2021). Alternative substrates can yield even higher efficiencies. Ethanol, for example, supports yields of up to 0.83 g_x_g_s_^−1^ for *C. jadinii* (Vieira‐Lara et al. 2024). Together, these values establish realistic performance benchmarks for microbial biomass production.

### 3.5 Safe for Consumption (GRAS)

Since microbial hydrolysates are intended for use in cultivated meat media, which will ultimately be consumed by humans, the source organisms should be classified as generally recognized as safe (GRAS) by the Food and Drugs Administration (FDA) in the United States of America (U.S. Food and Drug Administration n.d.), European Food Safety Authority or any other established food administration. Although the hydrolysate is not directly consumed, complete removal of microbial residues is time‐, cost‐, and energy intensive. Among bacteria, *B. subtilis* and *Corynebacterium glutamicum* have been granted GRAS status (GRAS notice no. 1143, and 792, respectively). Although the FDA approves some bacteria as production hosts for specific molecules, few are classified as GRAS in their entirety (Onyeaka et al. 2022). For cultivated meat applications, potential risks could be mitigated by washing the animal cells after culture, which reduces residual nucleic acids, toxins, and other microbial components prior to processing into the final product (Manning 2024).

Several yeasts are also considered GRAS. *S. cerevisiae* extracts are widely used in food and feed, both as flavor enhancer (monosodium glutamate substitute), and as nutritional yeast (e.g., Marmite, Vegemite). *C. jadinii* is another GRAS yeast, historically used as a protein supplement during World War I (Sousa‐Silva et al. 2021). Other yeasts proposed as SCP candidates include *Yarrowia lipolytica* (*Y. lipolytica*), *Candida tropicalis*, *K. marxianus*, *Komagataella phaffii* (*K. phaffi*, formerly known as *Pichia pastoris*), *Kluyveromyces marxianus*, and *Pichia kudriavzevii* (*Candida kruseii*) (Koukoumaki et al. 2024). Of these, *Y. lipolytica* (GRAS notice No. 940), *K. marxianus* (No. 88), and *K. phaffi* (No. 1219) have received GRAS notices for recombinant proteins or metabolite production. At the time of writing, *K. marxianus* biomass is the only organism of this list with a pending GRAS application for consumption (No. 1248).

### 3.6 Engineering Strategies Availability

Microorganisms can be engineered to recombinantly produce valuable compounds, with varying availability of genetic tools across species. Examples include increased production of specific amino acids (D'Este et al. 2018), vitamins (Abbas 2006; Branduardi et al. 2007), and growth factors (Lei et al. 2023). There are genomic engineering toolboxes available for *S. cerevisiae* (M. Zhao et al. 2024), *Y. lipolytica* (Larroude et al. 2019; M. X. Zhao et al. 2013), and methods have also been developed that apply across multiple species (*K. marxianus* (Rajkumar et al. 2019) and *Ogataea parapolymorpha* (Juergens et al. 2018)). These platforms provide potential for engineering the composition of the organism biomass, and thus of the microbial extract. A foreseen challenge would be retaining the amino acid of interest in the biomass (as part of the proteome) instead of producing the free amino acid, as is currently done in industry. Based on the analysis in Section 2, cysteine, glutamine, proline, and serine appear as likely targets.

## 4 Extracts

### 4.1 Extract Preparation Methods

Cells need to be lysed to make intracellular nutrients available, after which a mixture of soluble and insoluble compounds is obtained. Amino acids are present as free amino acids (< 200 Da) and as peptides in varying sizes. Protein fractionation showed most peptides from a yeast extract to be below 20 kDa (lower than 180 amino acid residues, approximately), followed by proteins ranging from 30 to 60 kDa, depending on the lysis method (Jacob et al. 2019). While the microorganism itself determines much of the extract's composition, the lysis method also plays a significant role. The lysis step determines to what extent nutrients can be extracted from the biomass. Common lysis methods used for microbial extract production are autolysis, plasmolysis, enzymatic hydrolysis, and physical disruption (Tao et al. 2023). Each method has its advantages and drawbacks, depending on the organism and application (see Table 2).

**Table 2:** Comparison of Different Production Methods of Yeast Extract Based on Their Performance in Several Criteria.

Methods	Yield	Nutrient retention	Cost and energy efficiency	Process efficiency	Scalability
Autolysis	−	−	+	−	+
Plasmolysis	+/−	+/−	+/−	−	−
Enzymatic degradation	+	+/−	− −	+/−	+/−
Physical disruption	+/−*	+	−	+	+

*Note:* Scorings are based on qualitative literature data from Tao et al. (2023) and Takalloo et al. (2020). Scores are color coded as follows: very low (− −): red; low (−): orange; moderate (+/−): yellow; high (+): green.

*A moderate score is given here due to variability between physical disruption methods.

Autolysis and plasmolysis are often used interchangeably in the scientific literature. We refer to autolysis as a process without additives that makes use of endogenous enzymes. In yeast, proteases, glucanases, and nucleases are activated during autolysis, typically by heat treatment at ~50°C for 24 h (Tanguler and Erten 2008). Plasmolysis combines autolysis with the use of additives to activate the endogenous enzymes and increase permeability of the cell wall, leading to higher protein yields (Takalloo et al. 2020). Common additives are ethanol, ethyl acetate, toluene, hexamethylenediamine, sodium chloride, Triton X‐100 detergent, diethyl ether, digitonin, and sucrose, of which ethanol, ethyl acetate, sodium chloride, sucrose, and saponins are food‐grade options (Berlowska et al. 2017; Tao et al. 2023). Some additives can inhibit microbial growth in a final product, thereby lowering contamination risks. However, introducing an organic solvent during lysis adds an extra purification step or implies the presence of the solvent in the extract. The toxicity of many organic solvents, specifically on cells relevant to cultivated meat, is not yet known. Thus, additives must be carefully screened for toxicity if used for microbial extract preparation. Enzymatic lysis uses exogenous enzymes such as proteases and glucanases, with known commercial products including Zymolyase, Flavourzyme, pancreatin, and Protamex (Tao et al. 2023). Enzymatic hydrolysis of *S. cerevisiae* has shown higher protein release than autolysis or plasmolysis (Takalloo et al. 2020). This method typically requires about 12 h, which is faster than autolysis and avoids high salt concentrations. However, the high cost of enzymes and the need for optimization remain a key limitation.

Physical disruption methods include high‐pressure homogenization, ultrasound, and bead milling (Tao et al. 2023). These methods are widely used in industry and are not species‐specific. A comparison between methods showed that 24 h plasmolysis led to a 98% yield based on the nitrogen released from the cells, whereas 2 h cell mill and sonotrode showed an average of 80% (Jacob et al. 2019). In contrast, the disruption of brewer's yeast (*Saccharomyces pastorianus*) with autolysis yielded < 18% protein, while the physical disruption with glass beads yielded between 43.5% and 47% protein (Vieira et al. 2013). However, the use of glass beads is only realistic in laboratories with small samples (< 100 g) (Shehadul Islam et al. 2017). Based on the literature, no method seems to consistently lead to the highest yield. Physical disruption methods are often performed using different machinery with varying settings, making it difficult to perform a conclusive, objective comparison. Autolysis and plasmolysis are straightforward and less costly in resource use, but enzymatic lysis is faster and might be more efficient in cleaving peptides. Physical disruption methods might need additional enzymatic breakdown of peptides for sufficient bioavailability of the extract.

### 4.2 Extract Bioavailability

When free amino acid concentrations are low, animal cells can utilize extracellular proteins as an alternative amino acid source through endocytic uptake and lysosomal degradation (Palm 2019; Palm et al. 2015). CHO cells expressing the PEPT1/PEPT2 peptide transporters can take up di‐ and tripeptides from the extracellular environment, which are subsequently hydrolyzed intracellularly to contribute to intracellular amino acid pools (Mosser et al. 2015; Naik et al. 2024). GlutaMAX™ (L‐alanyl‐L‐glutamine) is a dipeptide used to replace glutamine, an amino acid that is rapidly degraded in cultivation medium. Polypeptide mixtures of varying sizes promote growth in CHO cells, but there is still uncertainty about whether they contribute to cell nutrition or promote growth through other cellular mechanisms (Du et al. 2022; Lobo‐Alfonso et al. 2008; Mosser et al. 2013; Spearman et al. 2014, 2016). For example, ultrafiltration of a yeast extract demonstrated that most of the growth‐promoting effects on CHO cell culture could be attributed to the low molecular weight permeate fraction (< 10 kDa) (Mosser et al. 2015).

Peptide cleavage happens both extracellularly and intracellularly, meaning that larger peptides can be cleaved extracellularly before being taken up by the cells (Naik et al. 2024). Autolysis and enzymatic hydrolysis extracts can lead to 62%–81% of total peptides being < 1 kDa (< 10 amino acids), while high‐pressure homogenization and sonication produce 39%–76% (Oliveira et al. 2022). Physical lysis methods also showed up to 27% of peptides larger than 3 kDa, while autolysis and enzymatic hydrolysis led to fewer than 5% (Oliveira et al. 2022). Degradation of peptides by autolysis or enzymatic hydrolysis thereby aids in producing an extract with potentially higher bioavailability. Since this bioavailability is crucial for cell uptake, methods utilizing enzymatic breakdown (endogenous or added) of large peptides are better suited for this application. While cells can take up peptides of various sizes, the extent and mechanisms of this uptake remain poorly studied and warrant further investigation, for example, with ^13^C‐labeled microbial biomass.

### 4.3 Proliferation Assessment

To test the impact of a microbial extract on cultured cell growth, a proliferation assay is required. While yeast extract has been used extensively in CHO cell culturing as an additive (see Table 1), testing viability in cell lines relevant to cultivated meat has only recently gained attention. Growth inhibition occurred at concentrations of 100 mg/L in serum‐free medium (Dolgin et al. 2025), while no inhibition was seen with 1–5 g/L addition in medium containing 10% serum (Dong et al. 2023; Lei et al. 2023). The presence of FBS might therefore mitigate some of the negative effects of the added extract. Extracts in these studies were produced through physical lysis methods such as sonication and bead beating, which means they lacked the hydrolysis of proteins into small peptides and free amino acids. A low molecular weight fraction (< 3 kDa) of an autolyzed yeast extract showed a substantial increase in CHO cell growth, while the high molecular weight fractions (3–50 kDa) did not (Spearman et al. 2016). Hydrolyzed extracts might therefore elicit different toxicity responses and could therefore shift the range of concentrations that can be used.

The viability of extract‐supplemented cells should be assessed using an assay capable of screening multiple conditions in multi‐well plates, thereby avoiding labor‐intensive passaging and counting steps. Cell counting can be avoided by using dyes such as resazurin, which is converted into fluorescent resorufin by viable cells (Petiti et al. 2024). However, when components of the medium are replaced or reduced, a multi‐passage assay is required, as these subtle differences are not immediately apparent in single‐passage cell proliferation assays (Lyra‐Leite et al. 2023). Next to that, the ability of progenitor cells to differentiate into mature muscle or adipose cells is essential in cultivated meat, for accurate mimicry of taste, texture, and functionality (Lambert et al. 2024). The differentiation potential can be determined after incubation in differentiation medium and making use of immunofluorescence staining. Immunofluorescent staining images, and differentiation and fusion indexes are common ways to report differentiation potential (Stout et al. 2022, 2023).

Adherent proliferation assays are a suitable first screening tool, but growing cells in suspension is crucial for efficient and cost‐effective scale‐up of cultivated meat (Humbird 2021). It is also expected that the nutritional requirements of animal cells change when switching from adherent to suspension culture, as this was shown in other mammalian cell lines (Rybkowska et al. 2023). Microcarriers are often used in suspension growth as a surface for animal cells that usually grow in a monolayer (Bodiou et al. 2025). A different strategy is to grow cells in aggregates, where cells attach to neighboring cells (Klatt et al. 2024). Lastly, single‐cell suspension is a strategy that requires the cells not to attach to any surface (Pasitka et al. 2022). Obtaining a relevant suspension‐adapted cell line for academic research is challenging, as such lines are not widely available and often part of the intellectual property of companies. Additionally, adaptation to suspension growth is time consuming, inefficient and has a high failure rate (Contreras et al. 2026; Pasitka et al. 2025). Recently, the Good Food Institute acquired suspension‐adapted bovine cell lines from a cultivated meat startup that ceased operations and made them publicly available to support research and development in the field (Good Food Institute n.d.). In summary, testing extracts in proliferation assays is crucial, and suspension testing must be considered, as it is key to the successful scale‐up of cultivated meat.

## 5 Conclusion and Future Outlook

Cultivated meat could offer a promising alternative to traditional livestock products, but cell culture media costs remain a major challenge, specifically the contribution of highly purified individual amino acids. Compositional analysis shows that the amino acid profiles of traditional meat and cultivated meat largely overlap. A comparison between amino acid profiles of CHO cell composition, consumption profile, and the common basal medium DMEM/F‐12 highlights that cell amino acid uptake rates are the most suitable amino acid requirements for biomass production.

Next to amino acid composition compatibility, there are other factors to consider when choosing a microbial extract as amino acid replacement. Yeasts and bacteria are the most promising options due to their scalability, fast growth, and efficient nutrient conversion to extracts that are safe for consumption. Cell lysis and extraction methods include autolysis, plasmolysis, enzymatic lysis, and physical disruption, but the lack of standardization complicates comparison of results between studies and increases batch‐to‐batch variability. However, enzymatic degradation of large polypeptides, which is not possible in physical disruption methods, might be required to increase hydrolysate bioavailability. Furthermore, batch‐to‐batch variations of microbial extracts can be reduced by culturing microorganisms in controlled environments.

Most cultivated meat‐related amino acid consumption rates reported in the literature are derived from media that still contain FBS or lack absolute values expressed per cell dry weight. Most published data that comply to these criteria are only reported for CHO cell lines, given their extensive use in bioprocesses. Controlled continuous bioreactor cultivations enable the determination of cell line‐specific amino acid requirements, providing parameters essential for predicting scale‐up potential. In these controlled environments, regulated feed of amino acids (e.g., amino acid‐limitation) can be used to prevent cells from experiencing toxic concentrations. Moreover, collaboration in the field is needed to standardize reference cell lines, cultivated meat products, and further experimental methodology.

Using a microbial extract can reduce the high costs and energy demand associated with pharmaceutical‐grade individual amino acid production. Metabolic engineering is a potential route to improve titer, rate, or yield on glucose or alternative substrates, or to adapt the amino acid profile of a microorganism to better match the consumption requirements of animal cells. While genetic engineering could also be applied to cultivated meat cell lines (Riquelme‐Guzmán et al. 2024), for example, to decrease their amino acid needs or produce suspension phenotypes, it also complicates regulatory approval, as no cultivated meat products from genetically modified cell lines have received regulatory approval at the time of writing. Despite these challenges, cost‐effective replacement of conventional media components, such as amino acids, can drastically lower the raw material costs, thereby bringing cultivated meat closer to market viability.

## Author Contributions

**Jelle C. Hoek:** conceptualization, investigation, writing – original draft, writing – review and editing, visualization. **Jean‐Marc Daran:** writing – review and editing, supervision. **Marieke E. Klijn:** writing – review and editing, supervision, funding acquisition. **Marcel A. Vieira‐Lara:** writing – review and editing, supervision.

## Conflicts of interest

The authors declare no conflicts of interest.

## Supporting information

Supporting File 1

Supporting File 2

## Data Availability

The data that support the findings of this study are available in the supporting material of this article.
