# Supplementary material for: Microbial Hydrolysates as Amino Acid Source in Cell Culture Media for Cellular Agriculture
Source: Biotechnol Bioeng. 2026 Jul 3;123(10):2520–35. doi: 10.1002/bit.70290 (PMC13576808; doi:10.1002/bit.70290)
Supplement: Supplementary file 1 [file BIT-123-2520-s001.docx]

10. Supporting information
    10.1 Lyra-Leite et al. medium cost calculation

The optimized medium had a reduction of several components, but no cost calculation was performed in the original publication (Lyra-Leite et al., 2023). A cost calculation was performed based on 1 kg packages found on the Sigma-Aldrich website and is shown in tab 1 of supporting information file 2.

10.2 Humbird calculation

Humbird performed a comparison of U.S. soybean meal with total amino acid composition of CHO cells (Humbird, 2021). This calculation is repeated for *Saccharomyces cerevisiae*, *Cyberlindnera jadinii*, and *Bacillus subtilis* extracts in tab 3 of supporting information file 2.

10.3 Similarity between microbial extract amino acid compositions

**Figure S1. Amino acid fractions per amino acid for ten organisms.** Organisms have different symbols and are coloured by species. Full data is available in tab 5 of supporting information file 2.

10.4 S. cerevisiae amino acid composition comparison

Amino acid comparisons between species should be performed with caution, as amino acid compositions can differ significantly, even within the same species. Figure S2 compares the amino acid composition of CHO cellular protein to three different *Saccharomyces cerevisiae* extracts. Extract 1 was grown on glucose as carbon substrate, 2 on molasses, and 3 is commercially available yeast extract (also assumed to have grown on molasses).

**Figure S2. Comparison of amino acid profiles of extracts of Saccharomyces cerevisiae (S. cerevisiae).** 1 (Anderson et al., 2024), 2 (Demirgül et al., 2022), and 3 (BD Diagnostics, 2009). Hydrolysate values are normalized to the threonine amino acid content. Asn and Gln are grouped with Asp and Glu, respectively, due to the deamidation that occurs in acid protein hydrolysis protocols. Adapted from Figure 2, (Humbird, 2021). Full calculation of the data is available in tab 6 of supporting information file 2.

11. Supporting information references

Anderson, A., Van der Mijnsbrugge, A., Cameleyre, X., & Gorret, N. (2024). From yeast screening for suitability as single cell protein to fed-batch cultures. *Biotechnology Letters*, 1–16. https://doi.org/10.1007/S10529-024-03504-0/

BD Diagnostics. (2009). *Difco & BBL Manual of Microbiological Culture Media* (M. J. Zimbro, D. Power, S. M. Miller, G. E. Wilson, & J. A. Johnson, Eds.; 2nd ed.). BD Diagnostics - Diagnostic Systems.

Demirgül, F., Şimşek, Ö., Bozkurt, F., Dertli, E., & Sağdıç, O. (2022). Production and characterization of yeast extracts produced by *Saccharomyces cerevisiae*, *Saccharomyces boulardii* and *Kluyveromyces marxianus*. *Preparative Biochemistry & Biotechnology*, *52*(6), 657–667. https://doi.org/10.1080/10826068.2021.1983833

Humbird, D. (2021). Scale-Up Economics for Cultured Meat Techno-Economic Analysis and Due Diligence. *Biotechnology and Bioengineering*, *118*, 3239–3250. https://doi.org/10.1002/bit.27848

Lyra-Leite, D. M., Copley, R. R., Freeman, P. P., Pongpamorn, P., Shah, D., McKenna, D. E., Lenny, B., Pinheiro, E. A., Weddle, C. J., Gharib, M., Javed, H., Fonoudi, H., Sapkota, Y., & Burridge, P. W. (2023). Nutritional requirements of human induced pluripotent stem cells. *Stem Cell Reports*, *18*(6), 1371. https://doi.org/10.1016/J.STEMCR.2023.05.004
